# Effect of tolvaptan on the prognosis of patients with rapidly progressive autosomal dominant polycystic kidney disease: a prospective open-label study

**DOI:** 10.3389/fmed.2026.1739751

**Published:** 2026-03-12

**Authors:** Li Zhou, Wenge Li

**Affiliations:** Department of Nephrology, China–Japan Friendship Hospital, Beijing, China

**Keywords:** ADPKD, EGFR, renal, TKV, tolvaptan

## Abstract

**Introduction:**

Although tolvaptan is internationally recognized for its therapeutic benefits in autosomal dominant polycystic kidney disease (ADPKD) and has been referenced in the 2020 Chinese Clinical Practice Guidelines, it has yet to be approved in China due to limited large-scale local evidence.

**Methods:**

A two-year, prospective, single-center, open-label trial was conducted to assess the efficacy and safety of tolvaptan in patients with rapidly progressive ADPKD. The primary endpoint was the change in total kidney volume (TKV), while secondary endpoints included changes in eGFR and evaluation of safety and tolerability.

**Results:**

Eighty-nine patients were enrolled 80% had a family history of ADPKD, 40% had polycystic liver disease and 65.6% were diagnosed with hypertension. A proportion of patients also presented with renal insufficiency and vascular complications. Baseline laboratory tests showed a decreased median red blood cell count and elevated concentrations of urinary albumin, urea, creatinine and uric acid. The most commonly used daily doses of tolvaptan were 60 mg (31.1%) and 45 mg (26.7%). After treatment, the mean TKV change rate for the left kidney was −70.2 ± 206.07 mL at 6 months and −47.84 ± 132.58 mL at 12 months; for the right kidney, the respective changes were −42.69 ± 209.40 mL and −8.57 ± 286.86 mL. eGFR remained stable, with no major hepatic/renal toxicity.

**Conclusion:**

Tolvaptan effectively slowed kidney volume growth and was well tolerated in Chinese patients with rapidly progressive ADPKD.

## 1 Introduction

Autosomal dominant polycystic kidney disease (ADPKD) is the most common monogenic hereditary kidney disorder, affecting approximately 12.5 million individuals worldwide and is ranked as the fourth leading cause of end-stage kidney disease (ESKD) (1). According to data from the European Renal Association-European Dialysis and Transplant Association (ERA-EDTA), the prevalence of ADPKD in 19 European Union countries is estimated at 3.96 per 10,000 population (2). ADPKD is primarily characterized by progressive bilateral renal cyst growth and gradual renal function decline, with nearly 50% of patients reaching ESKD by around 60 years of age (3). Clinical manifestations include flank pain, abdominal distension, hematuria, proteinuria, nephrolithiasis, urinary tract infections and hypertension, along with extrarenal complications such as hepatic, pancreatic, splenic and arachnoid cysts, as well as intracranial aneurysms, mitral valve prolapse, diverticulosis, abdominal wall hernias and hyperlipidemia (4, 5).

Currently, there is no curative treatment for ADPKD, and clinical management mainly relies on supportive care, including blood pressure control-typically with angiotensin-converting enzyme inhibitors (ACEIs) or angiotensin II receptor blockers (ARBs)—as well as dietary restrictions on sodium and protein intake, avoidance of nephrotoxic agents, adequate hydration and symptomatic treatment. Renal replacement therapy is required after progression to ESKD.

Tolvaptan, a selective vasopressin V2 receptor antagonist remains the only pharmacological agent proven to delay ADPKD progression by inhibiting cyst growth, reflected by slowing TKV increase and a reduced rate of eGFR decline (6, 7). Landmark clinical trials, including TEMPO 3:4 (tolvaptan efficacy and safety in the management of ADPKD and its outcomes) and REPRISE trial (replicating evidence of preserved renal function: an investigation of tolvaptan safety and efficacy in ADPKD) have demonstrated its efficacy in patients with rapidly progressive ADPKD, leading to approval and guideline recommendations for the use of tolvaptan in the United States, Europe and Japan (8, 9).

One of the tools used to assess disease progression risk in ADPKD patients is the Mayo imaging-classification (MIC) model (10). This model integrates TKV measured by MRI or CT, the rate of TKV increase over time and the rate of eGFR decline to predict the risk of disease progression (5). The MIC model facilitates the identification of high-risk patients and serves as a crucial reference for clinical decision-making regarding tolvaptan therapy.

Although tolvaptan is widely used internationally, the 2020 Chinese Clinical Practice Guidelines for ADPKD have mentioned its therapeutic recommendation (11). However, due to the lack of large-scale clinical data in the Chinese population, tolvaptan has not yet been officially approved as a treatment for ADPKD in China, resulting in a very low usage rate, estimated to be <1%. Besides the indication restrictions, the high cost of the drug and concerns about diuretic-related adverse effects and hepatotoxicity also constitute major barriers to its widespread use. In practice, some medical centers have attempted low-dose tolvaptan regimens (e.g., 15/15 mg or 30/15 mg), and despite doses being lower than those recommended by international guidelines, favorable efficacy and tolerability have been observed (12).

Therefore, real-world studies are urgently needed to assess tolvaptan’s effectiveness and safety in Chinese ADPKD patients, with particular focus on how different dosing adjustment strategies impact the control of clinical symptoms, renal function preservation, long-term effects on liver function and serum sodium concentrations. By collecting continuous clinical data over 2 years, this study aimed to provide scientific evidence to support personalized tolvaptan therapy in a Chinese population and to facilitate its rational clinical use and policy approval.

## 2 Methods

This prospective, open-label, single-center clinical trial was conducted over 2 years. A total of 89 patients diagnosed with ADPKD were enrolled to represent a spectrum of disease stages (early, intermediate and advanced) and diverse clinical features including hypertension, renal impairment, and cyst burden. Tolvaptan was administered twice daily, with the first dose given at approximately 7:00 a.m. and the second dose approximately 8 h later. Treatment was initiated at 15 mg and gradually adjusted over 3–4 months based on each patient’s clinical condition and side effects until an individualized optimal dose was reached. For patients with prostate enlargement, nocturia was occasionally observed, and standard medications such as finasteride were prescribed when necessary. Medication adherence was evaluated during routine follow-up visits. Eligible patients were aged between 18 and 85 years, spanning Chronic Kidney Disease (CKD) stage 1–5, and had a confirmed clinical diagnosis of ADPKD supported by a clear family history and typical renal imaging findings characterized by enlarged kidneys with multiple cysts. All patients met the criteria for rapidly progressive ADPKD according to the Mayo Clinic imaging-classification (MIC) model, specifically classified as types 1C to 1E. Height-adjusted total kidney volume (HtTKV) was calculated as TKV divided by height (mL/m), where TKV (mL) was determined by the ellipsoid formula: *π*/6 × length × width × depth. Patients with an annual HtTKV growth rate (*α*) exceeding 3% were classified as MIC types 1C–1E. Estimated glomerular filtration rate (eGFR) was calculated using the CKD-EPI equations. Detailed formulas are provided in Appendix 1. Written informed consent was obtained from all patients before enrollment in the study.

### 2.1 Exclusion criteria

Patients were excluded from the study if they met any of the following criteria: known allergy or hypersensitivity to tolvaptan or its formulations; presence of abnormal liver function, hypovolemia or hypernatremia; pregnancy, breastfeeding or intention to become pregnant during the study period (within 18 months); inability to perceive thirst or maintain adequate oral hydration; diagnosis of advanced diabetes mellitus, renal carcinoma, solitary kidney, recent renal surgery, acute kidney injury or other severe renal diseases; participation in another clinical trial within the past 3 months; or any other condition deemed by the investigators to render the patient unsuitable for participation.

### 2.2 Study endpoints

The primary endpoint was to evaluate the effect of tolvaptan on TKV in patients with rapidly progressing ADPKD, measured as the difference between the expected and actual annualized growth rates of HtTKV at 6 and 12 months, based on Mayo imaging classification. Secondary endpoints included changes in eGFR from baseline at different time points during treatment, to evaluate the impact of tolvaptan on renal function. Safety and tolerability monitoring encompassed a detailed evaluation of all adverse events (AEs), including clinical symptoms, abnormal vital signs and abnormalities in laboratory parameters. Key safety indicators included liver function and renal function tests, complete blood counts and blood pressure. All AEs, serious adverse events (SAEs), and those deemed related to tolvaptan were recorded and evaluated in terms of type, severity, onset, duration, management, outcome and suspected causality.

### 2.3 Assessments

Patients were followed monthly for 24 months, with assessments conducted at baseline (Day 1) and at each follow-up visit. TKV was assessed using ultrasound every 3 to 4 months using the ellipsoid formula (length × width × depth × *π*/6). All measurements were performed by experienced sonographers who followed standardized protocols. Serum creatinine, eGFR, and serum sodium concentrations were measured at each visit during the study period. Given tolvaptan’s aquaretic effects, urinary parameters were also tracked, including the number of daytime and nighttime voids over 24 h at each time point. At each visit, investigators used non-leading questions actively to elicit information on adverse events. Laboratory evaluations performed at screening and/or baseline included alanine aminotransferase (ALT), aspartate aminotransferase (AST), total and direct bilirubin (TBIL, DBIL), total protein (TP), albumin (ALB), gamma-glutamyl transferase (GGT), alkaline phosphatase (ALP), blood urea nitrogen (Urea), serum creatinine (Cr), eGFR, potassium (K), and sodium (Na). All measurements were carefully documented for subsequent safety analysis.

### 2.4 Sample size estimation

This study was designed as a preliminary investigation into the efficacy and safety of tolvaptan in patients with rapidly progressing ADPKD. Based on the results of the TEMPO 3:4 trial, the average annual growth rate of TKV in the tolvaptan group was approximately 2.8%. Considering the characteristics of newly diagnosed ADPKD patients in real-world settings and limitations in research funding, the planned sample size for this study was 100 patients with rapidly progressing ADPKD.

### 2.5 Statistical analysis

All statistical analyses were performed using SPSS version 17.0 for Windows. Continuous variables with a normal distribution are expressed as the mean ± standard deviation (SD), and comparisons between groups were conducted using the independent-samples *t*-test. Categorical variables are presented as frequencies or percentages and were analyzed using the chi-squared (*χ*^2^) test. For continuous variables with a non-normal distribution, data are expressed as the median and interquartile range [M (IQR)], and intergroup comparisons were made using the Wilcoxon rank-sum test. A *p*-value <0.05 was considered to be statistically significant.

## 3 Results

Among the 89 patients with rapidly progressing ADPKD enrolled in this study, approximately 80% had a familial history of polycystic kidney, and 40% had co-existing polycystic liver disease. Regarding comorbidities, 65.6% of patients had hypertension, 15.6% had hyperlipidemia and kidney function varied among the cohort (detailed distribution by CKD stage is shown in Table 1). Additionally, 8.9% were diagnosed with arteriosclerosis obliterans and another 8.9% had deep vein thrombosis of the lower extremities (Table 1). Laboratory test results showed that the median red blood cell count was 3.0 × 10^12^/L (range: 1.20–8.50 × 10^12^/L), which was below the normal reference range. The median urine microalbumin concentration was 32.0 mg/L (range: 7.52–99.26 mg/L), exceeding the normal range. Serum urea and creatinine concentrations were generally elevated, and increased serum uric acid concentrations were also measured (Table 1).

**Table 1:** Baseline and clinical characteristics of patients.

Variable	Total (*n* = 89)	Reference
Age (years)	49.82 ± 12.38	Normal distribution
Median	52	
Min, max	23.0, 80.0	
Gender, *n* (%)		
Male	53 (60.0%)	
Female	36 (40.0%)	
BMI (kg/m^2^), median	23.17 ± 3.14	Normal distribution
Median	22.8	
Min, max	16.4, 34.6	
Inpatient diagnosis, *n* (%)		
Congenital polycystic kidney	72 (80.0%)	
Congenital polycystic liver	36 (40.0%)	
Hypertension	59 (65.6%)	
Lacunar cerebral infarction	3 (3.3%)	
Type 2 diabetes mellitus	2 (2.2%)	
Hyperlipidemia	14 (15.6%)	
Urea (mmol/L)	7.34 (4.82–11.96)	2.5–7.5
Cr (μmol/L)	146.20 (97.90–330.50)	45–110
eGFR (mL/min)	42.32 (14.43–73.33)	90–120
Chronic kidney disease stage		
Stage 1	12 (13.5%)	
Stage 2	22 (24.7%)	
Stage 3	30 (33.7%)	
Stage 4	4 (4.5%)	
Stage 5	21 (23.6%)	

ALB, albumin; ALP, alkaline phosphatase; ALT, alanine aminotransferase; AST, aspartate aminotransferase; CR, creatinine; DBIL, direct bilirubin; eGFR, estimated glomerular filtration rate; FER, ferritin; GGT, gamma-glutamyl transferase; GLU, glucose; HDL-C, high-density lipoprotein cholesterol; HGB, hemoglobin; LDL-C, low-density lipoprotein cholesterol; PLT, platelet; TBIL, total bilirubin; TC, total cholesterol; TG, triglycerides; TP, total protein; UA, uric acid.

In a further subgroup analysis, among 72 patients with congenital polycystic kidney disease, 36 with congenital polycystic liver disease and 35 with both conditions. The incidence of hypertension ranged from 77.8 to 86.1%, while the incidence of hyperlipidemia ranged from 18.1 to 22.2%. Moreover, abdominal distension and pain was reported in 13.9% of patients with polycystic kidney disease, 22.2% with polycystic liver disease and 22.9% with both conditions (Table 2). These findings suggest a potential association between the involvement of different cystic organs and the development of hypertension, renal impairment and vascular-related complications, indicating the need for more tailored management strategies in this patient population.

**Table 2:** Comorbidities and clinical characteristics in patients with congenital polycystic kidney and/or liver disease.

	Congenital polycystic kidney (*n* = 72)	Congenital polycystic liver (*n* = 36)	Congenital polycystic kidney + polycystic liver (*n* = 35)
Comorbidities			
Edema	2 (2.8)	2 (5.6)	2 (5.7)
Hypertension	56 (77.8)	31 (86.1)	30 (85.7)
Lacunar cerebral infarction	2 (2.8)	3 (8.3)	2 (5.7)
Type 2 diabetes mellitus	2 (2.8)	2 (5.6)	2 (5.7)
Hyperlipidemia	13 (18.1)	8 (22.2)	7 (20.0)
Chronic renal insufficiency	11 (15.3)	4 (11.1)	4 (11.4)
Arteriosclerosis obliterans	8 (11.1)	2 (5.6)	2 (5.7)
Deep vein thrombosis of the lower extremities	6 (8.3)	3 (8.3)	3 (8.6)
Clinical characteristics			
Abdominal distention and pain	10 (13.9)	8 (22.2)	8 (22.9)
Gastroesophageal reflux	2 (2.8)	0 (0.0)	0 (0.0)
Insomnia	3 (4.2)	0 (0.0)	0 (0.0)

**Figure 1:**
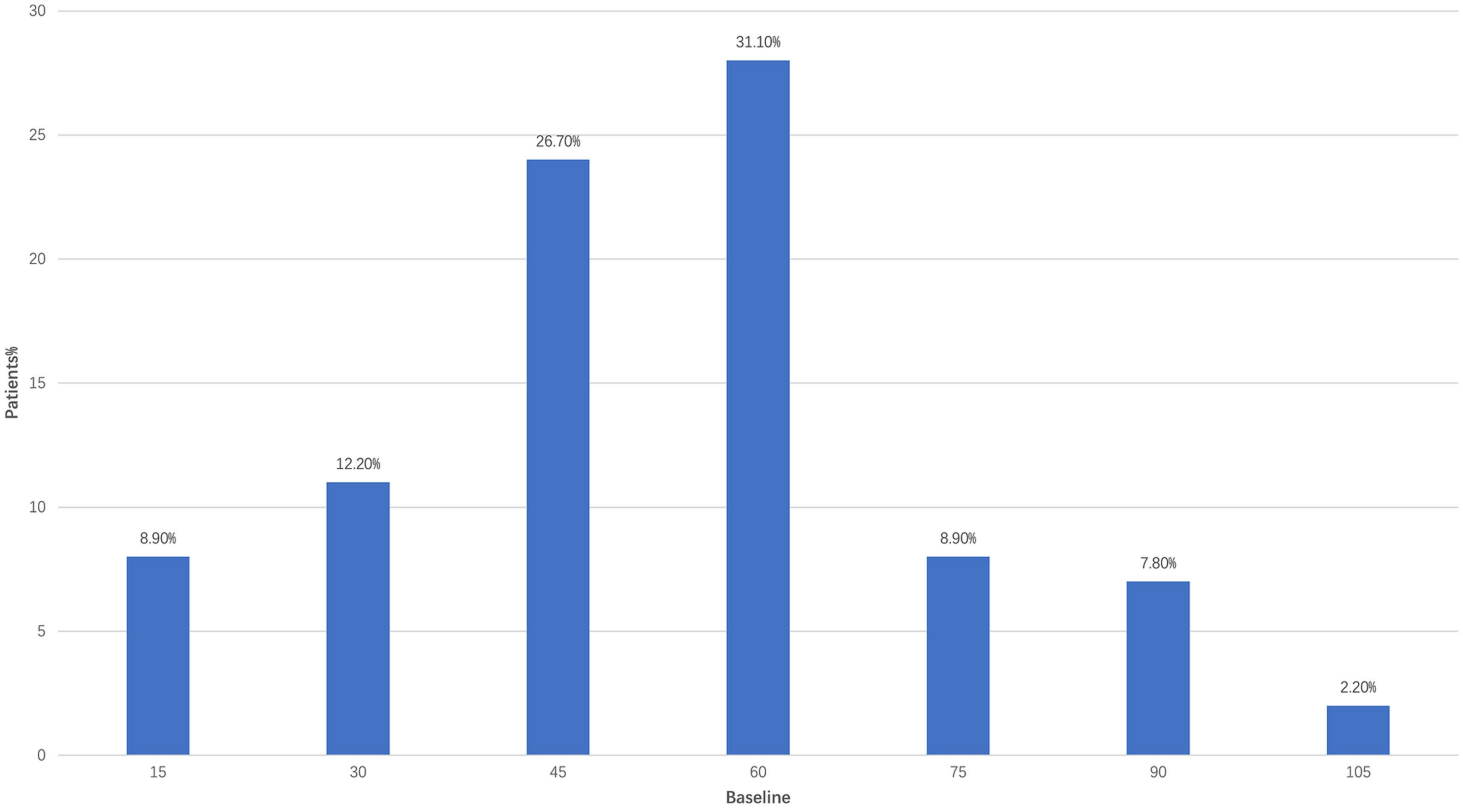
Distribution of patients by tolvaptan dosage.

Regarding medication usage, among the enrolled ADPKD patients, the most frequently prescribed dose of tolvaptan was 60 mg (31.1%), followed by 45 mg (26.7%) and 30 mg (12.2%) (Figure 1). In terms of therapeutic efficacy, as shown in Table 3, tolvaptan treatment demonstrated notable improvements in TKV. The change rate of TKV from baseline was −127.15 ± 302.74 at 6 months and −50.34 ± 325.07 mL at 12 months, indicating overall stabilization or modest reduction in kidney volume over the observation period. Regarding the volume of the largest renal cyst, the left kidney showed changes of −8.1 ± 33.45 at 6 months and −5.04 ± 13.65 at 12 months, while the right kidney showed changes of −8.94 ± 22.11 and −4.42 ± 12.39, respectively (Table 4).

**Table 3:** Changes in eGFR over time during tolvaptan treatment.

Time point (month)	eGFR (mL/min)	Difference (value − baseline)
Baseline	42.32 (14.43–73.33)	
1 month	40.16 (17.39–77.43)	−1.42 (−4.22 to 2.93)
6 months	39.72 (19.79–83.00)	−0.68 (−4.52 to 6.72)
12 months	39.84 (36.79–82.99)	−0.52 (−6.00 to 22.36)
18 months	41.30 (30.00–78.40)	0.94 (−2.50 to 17.67)
24 months	35.00 (25.35–53.51)	−3.53 (−9.66 to 19.33)

**Table 4:** Tolvaptan-induced changes in kidney volumes and TKV rate.

Structure	Volume			TKV rate	
	Baseline (mL)	6 months (mL)	12 months (mL)	6 months	12 months
Left kidney	3424.59 ± 3573.57	2648.19 ± 2166.37	2851 ± 3.398.35	−70.2 ± 206.07	−47.84 ± 132.58
Right kidney	3268.26 ± 2823.05	2597.19 ± 2030.15	2510.75 ± 2049.02	−42.69 ± 209.40	−8.57 ± 286.86
Both kidney	6682.27 ± 6117.73	5270.62 ± 4186.45	5409.13 ± 4666.93	−127.15 ± 302.74	−50.34 ± 325.07
Left kidney largest cyst	237.08 ± 514.13	163.79 ± 173.23	131.19 ± 190.77	−8.1 ± 33.45	−5.04 ± 13.65
Right kidney largest cyst	196.04 ± 258.15	138.65 ± 120.32	126.9 ± 150.45	−8.94 ± 22.11	−4.42 ± 12.39

Similarly, as shown in Table 3, tolvaptan appeared to have a positive impact on eGFR, with improvements observed as the dose increased and the treatment duration extended to 1 year. Notably, eGFR values showed marked improvement compared to baseline during the early treatment phase. While some decline was noted over time, tolvaptan demonstrated a potential renal-protective effect, with the mean eGFR decreased from 42.32 mL/min/1.73 m^2^ at baseline to 39.84 mL/min/1.73 m^2^ at 12 months, corresponding to an annual decline of −0.8484 ± 1.817 mL/min/1.73 m^2^/year. At 24 months, the mean eGFR was 35.00 mL/min/1.73 m^2^, with an annual decline of −0.8672 ± 1.131 mL/min/1.73 m^2^.

**Figure 2:**
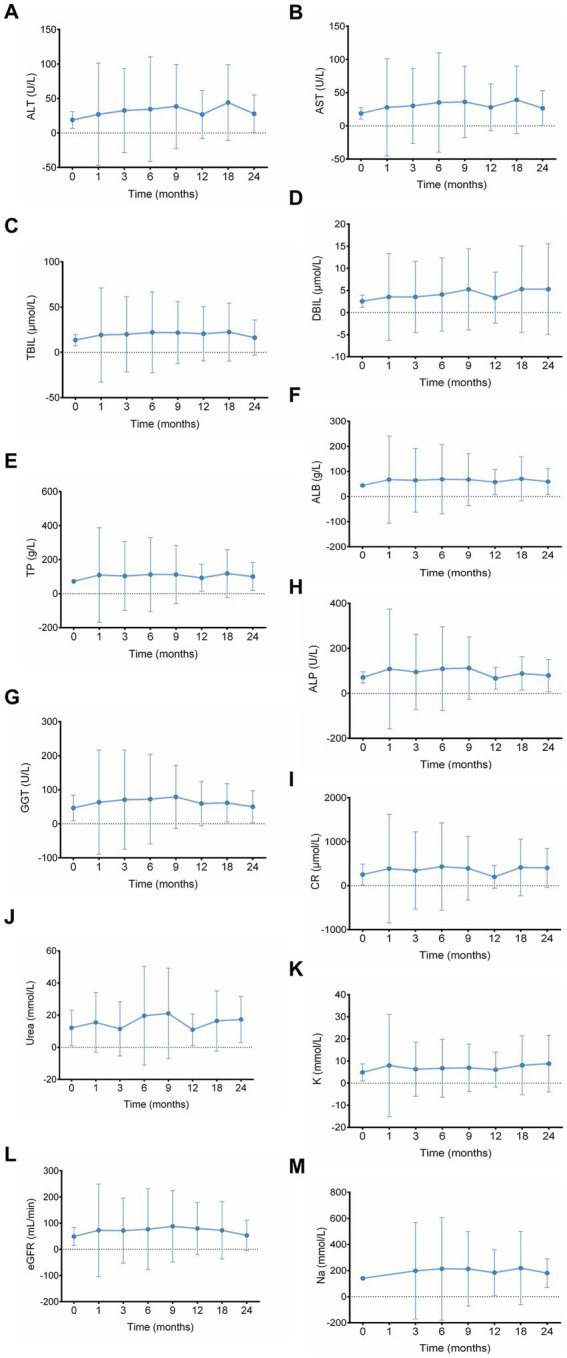
Evaluation of the long-term effects of tolvaptan on eGFR, liver function, renal function, and related parameters. **(A)** ALT, **(B)** AST, **(C)** TBIL, **(D)** DBIL, **(E)** TP, **(F)** ALB, **(G)** GGT, **(H)** ALP, **(I)** Cr, **(J)** urea, **(K)** K, **(L)** eGFR, and **(M)** Na levels were monitored at baseline and at 1, 3, 6, 9, 12, 18, and 24 months. Data are presented as mean ± SD. ALT, alanine aminotransferase; AST, aspartate aminotransferase; TBIL, total bilirubin; DBIL, direct bilirubin; TP, total protein; ALB, albumin; GGT, gamma-glutamyl transferase; ALP, alkaline phosphatase; Cr, creatinine; K, potassium; Na, sodium; eGFR, estimated glomerular filtration rate.

Moreover, no significant adverse effects on renal function, liver function, or eGFR were observed during the follow-up period with long-term tolvaptan use (Figure 2).

## 4 Discussion

This study is the first to assess the real-world effectiveness and safety of tolvaptan over a two-year period in a Chinese population with ADPKD. The results showed that tolvaptan significantly slowed the rate of TKV increase. At both 6 and 12 months of treatment, TKV in both the left and right kidneys decreased significantly (*p* < 0.05). Although eGFR as a secondary endpoint did not show a statistically significant change at 12 months, its overall trajectory remained stable during the treatment period without a sustained decline, suggesting a potential renoprotective effect of tolvaptan. The results support the proposed mechanism of action of tolvaptan as a vasopressin V2 receptor antagonist that inhibits cAMP-mediated cystogenesis (13). Elevated intracellular cAMP is a key driver of cyst initiation and expansion in ADPKD (14). By blocking V2 receptor signaling, tolvaptan attenuates disease progression at its upstream regulatory node (14).

These observations are consistent with findings from the TEMPO 3:4 trial, which reported a reduction in the annual eGFR decline from −3.70 to −2.72 mL/min/1.73 m^2^ following tolvaptan treatment (8). A similar trend was observed in the present study, with the mean annual eGFR decline of −0.8484 mL/min/1.73 m^2^ in the first year and −0.8672 mL/min/1.73 m^2^ in the second year. Supporting the potential renoprotective effect of tolvaptan in Chinese patients with ADPKD. The transient elevation in eGFR observed at 12 months may reflect improved volume status or measurement variability and warrants further investigation in future prospective studies.

In our cohort, patients spanned CKD stages 1–5, and TKV decreased slightly from 6682.27 ± 6117.73 mL at baseline to 5409.13 ± 4666.93 mL at 12 months, corresponding to a mean change of −50.34 ± 325.07 mL, indicating overall stabilization of kidney volume with a slight decrease in some patients. By comparison, the TEMPO 3:4 trial primarily enrolled patients with mean eGFR at 81.35 ± 21.02 mL/min/1.73 m^2^, in whom tolvaptan slowed TKV growth to approximately 2.8%/year (8). The inclusion of patients with more advanced CKD in our cohort may explain why the mean TKV showed a small decline, consistent with the known effect of tolvaptan in stabilizing kidney volume.

Although TKV growth serves as a key surrogate marker for ADPKD progression, changes in eGFR should be interpreted cautiously, as they may not be solely attributable to the cyst burden. Comorbid conditions such as hypertension and hyperlipidemia, observed in some patients, could also impact eGFR. Therefore, comprehensive renal imaging—preferably magnetic resonance imaging (MRI)—in conjunction with Mayo classification is recommended to assess the risk of disease progression.

Although an annual TKV increase of ≥5% has been proposed as an indicator of rapid progression, this requires at least three serial imaging evaluations, which can be challenging in routine clinical settings. Moreover, volumetric measurements are susceptible to variation due to differences in imaging equipment, parameters and operator technique. In comparison, a single MRI-based kidney volume assessment combined with Mayo classification offers a more practical and reliable approach to risk stratification in real-world clinical practice.

Tolvaptan demonstrated good safety and tolerability in the present study, with no cases of transaminase elevation or drug-induced liver injury observed. These findings are consistent with previous studies conducted both domestically and internationally, suggesting that tolvaptan also exhibits favorable hepatic safety in Asian populations. For example, meta-analyses of the TEMPO and REPRISE trials in patients with ADPKD reported that the incidence of ALT or AST elevation was approximately 2–3%, with most cases being mild and transient. Serious liver injury meeting Hy’s law criteria was extremely rare (15, 16). These findings suggest that, with regular liver function monitoring, tolvaptan exhibits an acceptable hepatic safety profile in clinical use. In addition, tolvaptan exerts its aquaretic effect by selectively antagonizing the vasopressin V2 receptor, promoting the excretion of free water without significantly affecting serum sodium or potassium concentrations. As a result, it poses a lower risk of electrolyte imbalances and renal function deterioration (16).

In line with its aquaretic activity, the most common AEs observed were increased urine output and urinary frequency, particularly in patients with preserved renal function. These effects typically peaked during the first week of treatment and then gradually subsided. A small number of patients experienced transient elevations in serum creatinine or orthostatic hypotension, likely attributable to fluid loss under insufficient hydration.

Importantly, no patients in the present study discontinued treatment due to aquaresis-related discomfort. With appropriate fluid and dietary management and individualized dose titration, most patients adapted well to the treatment. These results underscore that, while aquaretic effects are expected, they are generally predictable and manageable in clinical practice.

Despite promising results, this single-center, non-randomized study is subject to confounding factors, with a small sample size and the short follow-up limiting assessment of long-term outcomes and safety. Real-world variations in dosing and adherence may have affected treatment consistency. In addition, total kidney volume (TKV) was assessed using abdominal ultrasound, which is widely available, and feasible for repeated measurements in a high-volume clinical setting. While ultrasound provides reliable estimation of kidney size and overall cyst burden, it lacks fine structural resolution of MRI or CT, such as detecting small cysts or subtle morphological changes. These limitations may slightly affect the accuracy of risk stratification, but ultrasound remains a practical tool for longitudinal monitoring in real-world clinical practice. A potential limitation of this exploratory study in a Chinese patient population is that treatment was initiated at a lower dose than the KDIGO 2025 guideline-recommended starting dose, and future studies are needed to evaluate the impact of higher doses on patient outcomes and safety. In the Chinese population, tolvaptan may be effective at doses lower than internationally recommended for slowing kidney enlargement and cyst growth, but supporting evidence remains limited. Additionally, the high cost and lack of approved use in China have restricted access and optimal dosing, potentially impacting treatment efficacy. Larger, multi-center studies with standardized protocols and longer follow-up times are needed to validate these findings and inform region-specific treatment strategies.

## 5 Conclusion

This present study clearly demonstrated that tolvaptan significantly slowed kidney volume growth in Chinese patients with rapidly progressing ADPKD, with eGFR remaining stable and maintained over 2 years of treatment. No significant hepatic or renal toxicity was observed, indicating a favorable safety profile. These findings provide important clinical evidence supporting the efficacy and safety of tolvaptan in a Chinese ADPKD population and highlight its potential as a viable therapeutic option for patients with rapid disease progression.

## Data Availability

The original contributions presented in the study are included in the article/Supplementary material, further inquiries can be directed to the corresponding author.
