# Supplementary material for: Effect of tolvaptan on the prognosis of patients with rapidly progressive autosomal dominant polycystic kidney disease: a prospective open-label study
Source: Front Med (Lausanne). 2026 Mar 12;13:1739751. doi: 10.3389/fmed.2026.1739751 (PMC13017235; doi:10.3389/fmed.2026.1739751)
Supplement: Supplementary file 1 [file Table_1.DOCX]

Appendix 1

| Biomarkers Available | Sex | Condition | eGFR Equation |
| --- | --- | --- | --- |
| Serum creatinine only | Female | Cr ≤ 61.88 µmol/L | 142 × (Cr/61.88)^(-0.241) × 0.9938^Age × 1.012 |
|  |  | Cr > 61.88 µmol/L | 142 × (Cr/61.88)^(-1.200) × 0.9938^Age × 1.012 |
|  | Male | Cr ≤ 79.56 µmol/L | 142 × (Cr/79.56)^(-0.302) × 0.9938^Age |
|  |  | Cr > 79.56 µmol/L | 142 × (Cr/79.56)^(-1.200) × 0.9938^Age |
| Serum creatinine + cystatin C | Female | Cr ≤ 61.88 µmol/L, Cys C ≤ 0.8 mg/L | 135 × (Cr/61.88)^(-0.219) × (Cys C/0.8)^(-0.323) × 0.9961^Age × 0.963 |
|  |  | Cr ≤ 61.88 µmol/L, Cys C > 0.8 mg/L | 135 × (Cr/61.88)^(-0.219) × (Cys C/0.8)^(-0.778) × 0.9961^Age × 0.963 |
|  |  | Cr > 61.88 µmol/L, Cys C ≤ 0.8 mg/L | 135 × (Cr/61.88)^(-0.544) × (Cys C/0.8)^(-0.323) × 0.9961^Age × 0.963 |
|  |  | Cr > 61.88 µmol/L, Cys C > 0.8 mg/L | 135 × (Cr/61.88)^(-0.544) × (Cys C/0.8)^(-0.778) × 0.9961^Age × 0.963 |
|  | Male | Cr ≤ 79.56 µmol/L, Cys C ≤ 0.8 mg/L | 135 × (Cr/79.56)^(-0.144) × (Cys C/0.8)^(-0.323) × 0.9961^Age |
|  |  | Cr ≤ 79.56 µmol/L, Cys C > 0.8 mg/L | 135 × (Cr/79.56)^(-0.144) × (Cys C/0.8)^(-0.778) × 0.9961^Age |
|  |  | Cr > 79.56 µmol/L, Cys C ≤ 0.8 mg/L | 135 × (Cr/79.56)^(-0.544) × (Cys C/0.8)^(-0.323) × 0.9961^Age |
|  |  | Cr > 79.56 µmol/L, Cys C > 0.8 mg/L | 135 × (Cr/79.56)^(-0.544) × (Cys C/0.8)^(-0.778) × 0.9961^Age |

Note：’Age is expressed in years; Cr = serum creatinine (µmol/L); Cys C = cystatin C (mg/L); 0.7 mg/dL = 61.88 µmol/L; 0.9 mg/dL = 79.56 µmol/L.
